# Structural and functional analysis of four non-coding Y RNAs from Chinese hamster cells: identification, molecular dynamics simulations and DNA replication initiation assays

**DOI:** 10.1186/s12867-015-0053-5

**Published:** 2016-01-05

**Authors:** Quirino Alves de Lima Neto, Francisco Ferreira Duarte Junior, Paulo Sérgio Alves Bueno, Flavio Augusto Vicente Seixas, Madzia Pauline Kowalski, Eyemen Kheir, Torsten Krude, Maria Aparecida Fernandez

**Affiliations:** Departamento de Biotecnologia, Genética e Biologia Celular, Universidade Estadual de Maringá, Av. Colombo 5790, Maringá, Paraná 87020-900 Brazil; Departamento de Bioquímica, Universidade Estadual de Maringá, UEM, Paraná, 87020-900, Brazil; Department of Zoology, University of Cambridge, Downing Street, Cambridge, CB2 3EJ UK

**Keywords:** Non-coding RNAs, Chinese hamster Y RNAs, DNA replication, Nucleic acid simulations

## Abstract

**Background:**

The genes coding for Y RNAs are evolutionarily conserved in vertebrates. These non-coding RNAs are essential for the initiation of chromosomal DNA replication in vertebrate cells. However thus far, no information is available about Y RNAs in Chinese hamster cells, which have already been used to detect replication origins and alternative DNA structures around these sites. Here, we report the gene sequences and predicted structural characteristics of the Chinese hamster Y RNAs, and analyze their ability to support the initiation of chromosomal DNA replication in vitro.

**Results:**

We identified DNA sequences in the Chinese hamster genome of four Y RNAs (chY1, chY3, chY4 and chY5) with upstream promoter sequences, which are homologous to the four main types of vertebrate Y RNAs. The *chY1*, *chY3* and *chY5* genes were highly conserved with their vertebrate counterparts, whilst the *chY4* gene showed a relatively high degree of diversification from the other vertebrate *Y4* genes. Molecular dynamics simulations suggest that chY4 RNA is structurally stable despite its evolutionarily divergent predicted stem structure. Of the four *Y* RNA genes present in the hamster genome, we found that only the *chY1* and *chY3* RNA were strongly expressed in the Chinese hamster GMA32 cell line, while expression of the *chY4* and *chY5* RNA genes was five orders of magnitude lower, suggesting that they may in fact not be expressed. We synthesized all four chY RNAs and showed that any of these four could support the initiation of DNA replication in an established human cell-free system.

**Conclusions:**

These data therefore establish that non-coding chY RNAs are stable structures and can substitute for human Y RNAs in a reconstituted cell-free DNA replication initiation system. The pattern of Y RNA expression and functionality is consistent with Y RNAs of other rodents, including mouse and rat.

**Electronic supplementary material:**

The online version of this article (doi:10.1186/s12867-015-0053-5) contains supplementary material, which is available to authorized users.

## Background

For many years, the most well-studied sequences in the human genome have been those of protein-coding genes. Nevertheless, most of the genome is transcribed as non-coding RNA (ncRNA) and is never translated into protein [1]. It has become increasingly apparent that ncRNA is crucially important for a wide array of cellular functions [2, 3].

The class of small non-coding RNAs termed Y RNAs have a function as essential factors for the initiation of chromosomal DNA replication in mammalian somatic cells [4]. Y RNAs have originally been described as the RNA component of Ro ribonucleoprotein particles (Ro RNPs), which contain proteins Ro60 and La and are detected by autoimmune antibodies from patients suffering from systemic lupus erythematosus [5, 6]. Despite their relatively small size, Y RNAs are involved in several independent cellular pathways, including RNA surveillance and RNA quality control, in addition to DNA replication [4, 7–10]. Y RNAs have been shown to biochemically interact and co-localize with several proteins that are essential for the initiation of DNA replication, including the origin recognition complex, ORC [11].

The individual *Y* RNA genes are located in close proximity to each other in vertebrate genomes, including human, mouse and *Xenopus* [12]. The RNA polymerase III transcribes each gene from an upstream class 3 promoter. There are four distinct Y RNAs in humans (hY1, hY3, hY4 and hY5) and only two Y RNAs in mice (mY1 and mY3) and other rodents, where they all range in size from 70 to 115 nucleotides (nt) [12, 13]. All Y RNAs form characteristic stem − loop structures, which are due to partially complementary 5′ and 3′ domains that form the lower and upper stems with a large internal loop [9, 10, 14, 15]. The highly conserved upper stem domain of vertebrate Y RNAs is essential and sufficient for their DNA replication initiation function, due to the presence of a functionally essential GUG-CAC trinucleotide motif [9, 10, 14, 15]. However, the molecular mechanism underpinning this function is currently unknown.

Cells of the Chinese hamster (*Cricetulus griseus*) have become an important model to study metazoan DNA replication, in particular lung fibroblasts that were selected for overproduction of adenylate deaminase 2 (AMPD2) due to local gene amplification by a treatment with coformycin [16]. In an amplified locus containing the *AMPD2* and other genes, the ori*GNAI3* DNA replication origin was detected by 2D gel electrophoresis and competitive PCR replicon mapping techniques [17, 18]. By means of the dynamic molecular combing procedure, it was possible to map three more DNA replication origins (ori*C*, ori*B*, and ori*A*) in that polygenic region [19]. Additionally, these DNA replication origins co-localize with A + T rich regions identified as matrix attachment regions [20]. Our laboratory has recently identified, through in silico and circular permutation analysis, that these DNA replication origin sequences are situated in nucleosome-free regions and are associated with intrinsically bent DNA segments [21, 22]. Therefore, Chinese hamster cells are an excellent model system for analyzing chromosomal DNA replication at a local level. However, Y RNAs that may play an essential role in this process have not been described in this organism to date. Therefore, we have searched for homologs of human *Y* RNA genes in the genome of this rodent.

Here we report the identification of four genes coding for Chinese hamster Y RNAs (*chY1*, *chY3*, *chY4* and *chY5* RNAs). We have characterized the predicted secondary structures of chY RNAs and analyzed the expression of chY RNAs in Chinese hamster cells. We have tested whether synthetic chY RNAs can functionally substitute the human Y RNAs in a cell-free DNA replication system. Lastly, since chY4 RNA has an evolutionarily divergent secondary structure in the upper stem from other vertebrate Y RNAs, we have carried out molecular dynamics simulation analysis to investigate whether this segment is expected to be stable at physiological conditions.

## Results and discussion

### Homology search and predicted secondary structures

**Fig. 1:**
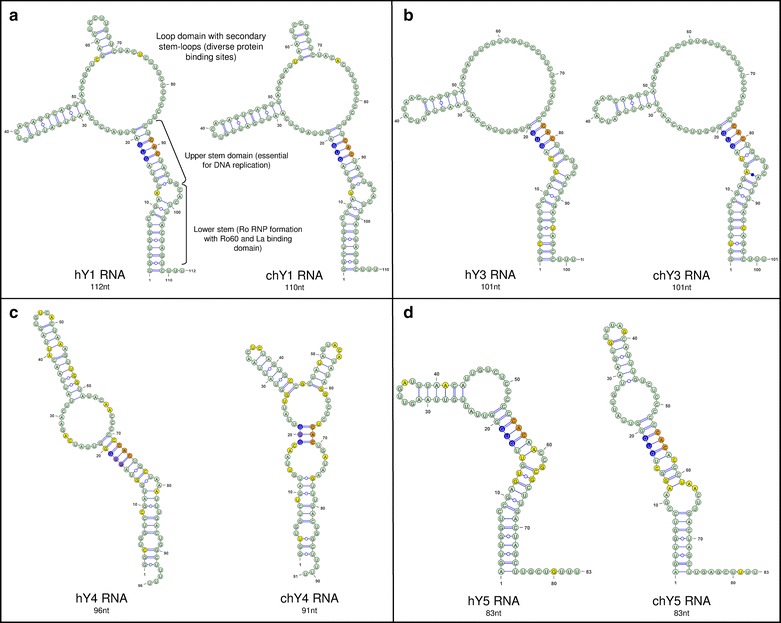
Nucleotide sequences and predicted secondary structures of human and Chinese hamster Y RNAs. Comparison between hY1 and chY1 RNAs (**a)**, hY3 and chY3 RNAs (**b**), hY4 and chY4 RNAs (**c**), and hY5 and chY5 RNAs (**d**). *Black brackets* indicate locations of conserved structural elements, with their known functions described alongside. Essential for DNA replication, the GUG-CAC sequence motif is highlighted in *blue* and *orange* respectively. The divergent nucleotides between hY and chY RNAs are shown in *yellow*

After performing a homology search in the Chinese hamster genome, we found four candidate genes that could be homologs of human *Y* RNAs. These genes received an annotation and can be accessed at GenBank by codes: [JX559781.1] *chY1*, [JX976178.1] *chY3*, [JX976179.1] *chY4*, and [JX976180.1] *chY5* (Additional file 1: Figure S1). In addition to the gene body, these *chY* RNA genes had signatures for promoter and terminator elements for transcription by RNA polymerase III (Additional file 2: Figure S2). Nucleotide sequences and predicted secondary structures of Y RNAs are conserved within vertebrates [9, 12, 13, 23]. The sizes of all four chY RNAs are similar to their homologous hY RNAs. The chY1, chY3 and chY5 RNAs feature all the expected structural motifs of the corresponding hY RNA secondary structures, including the Ro60-binding lower stem, the DNA replication-promoting upper stem and a heterologous central loop (Fig. 1). In contrast, chY4 RNA has a shorter upper stem and a bigger loop between stems than the hY4 RNA (Fig. 1). At the nucleotide sequence level, the chY1 and chY3 RNAs have a high degree of similarity with their human homologues, while chY4 and chY5 are less conserved (Additional file 1: Figure S1).

Genes coding for *Y* RNAs are evolutionarily conserved in vertebrates, though different numbers of *Y* RNA genes exist in different species due to gene losses, duplications and rearrangements [12, 13, 23, 24]. In different rodent lineages, *Y1* and *Y3* genes are highly conserved and expressed, whereas *Y4* and *Y5* genes are often lost, or only present in the genome as remnant ‘fossil genes’ at their conserved syntenic locations (Additional file 3: Figure S3) [12, 13, 25, 26]. Therefore, we investigated next whether all four *chY* RNA genes are expressed in Chinese hamster cells.

### Expression analysis of chY RNAs

We determined the relative expression levels for each of the four chY RNAs by quantitative real-time PCR. To standardize chY RNA expression levels, we normalized the qPCR data to chY3 RNA, which showed the highest relative expression level. The results are shown in Table 1.

**Table 1:** chY RNA expression analysis

chY RNA	Relative Y RNA amount (n)	Standard deviation
chY1	0.93	±0.18
chY3	1	±0.20
chY4	2.37 × 10^−5^	±3.88 × 10^−6^
chY5	5.91 × 10^−5^	±1.75 × 10^−5^

Mean values and standard deviations of the chY RNA expression analysis from 3 to 4 independent experiments are shown. (n) = relative expression levels of each chY RNA, normalized to the amount of chY3 RNA molecules

Of the four genes coding for *Y* RNAs that are present in the Chinese hamster genome, only chY1 and chY3 RNAs are expressed to high and mutually comparative levels in the GMA32 cell line. In contrast, chY4 and chY5 RNA expression levels were detected at levels between four to five orders of magnitude below those of chY1 and chY3 RNAs (Table 1), indicating that they may be minimally expressed, if at all. Therefore, the situation in Chinese hamster cells is similar to other rodents, where Y4 and Y5 RNAs are not detectably expressed, and the corresponding genomic sequences are considered as “fossil” genes [12, 13]. This could explain the level of nucleotide divergence between the Chinese hamster and the human Y4 and Y5 RNAs. However, a sequence analysis of the promoter regions of the *Y5* RNA genes of *Homo sapiens*, *C. griseus* and *Mus musculus* revealed the presence of all expected type 3 promoter elements recognized by RNA polymerase III, with a high level of conservation (Additional file 2: Figure S2) [27–29]. This preservation of functional promoter elements therefore suggests that other factors are likely regulating the expression of rodent Y RNAs, such as epigenetic modifications or post-transcriptional degradation.

Within each species, and even across vertebrate species boundaries, individual Y RNAs show functional redundancy with each other as DNA replication factors in vitro [8]. Therefore, it is conceivable that in order to compensate for the absence of Y4 and Y5 RNA expression, the expression of Y1 and Y3 RNAs may be upregulated in rodents compared to human and other species expressing more than two Y RNAs.

### DNA replication in vitro

**Fig. 2:**
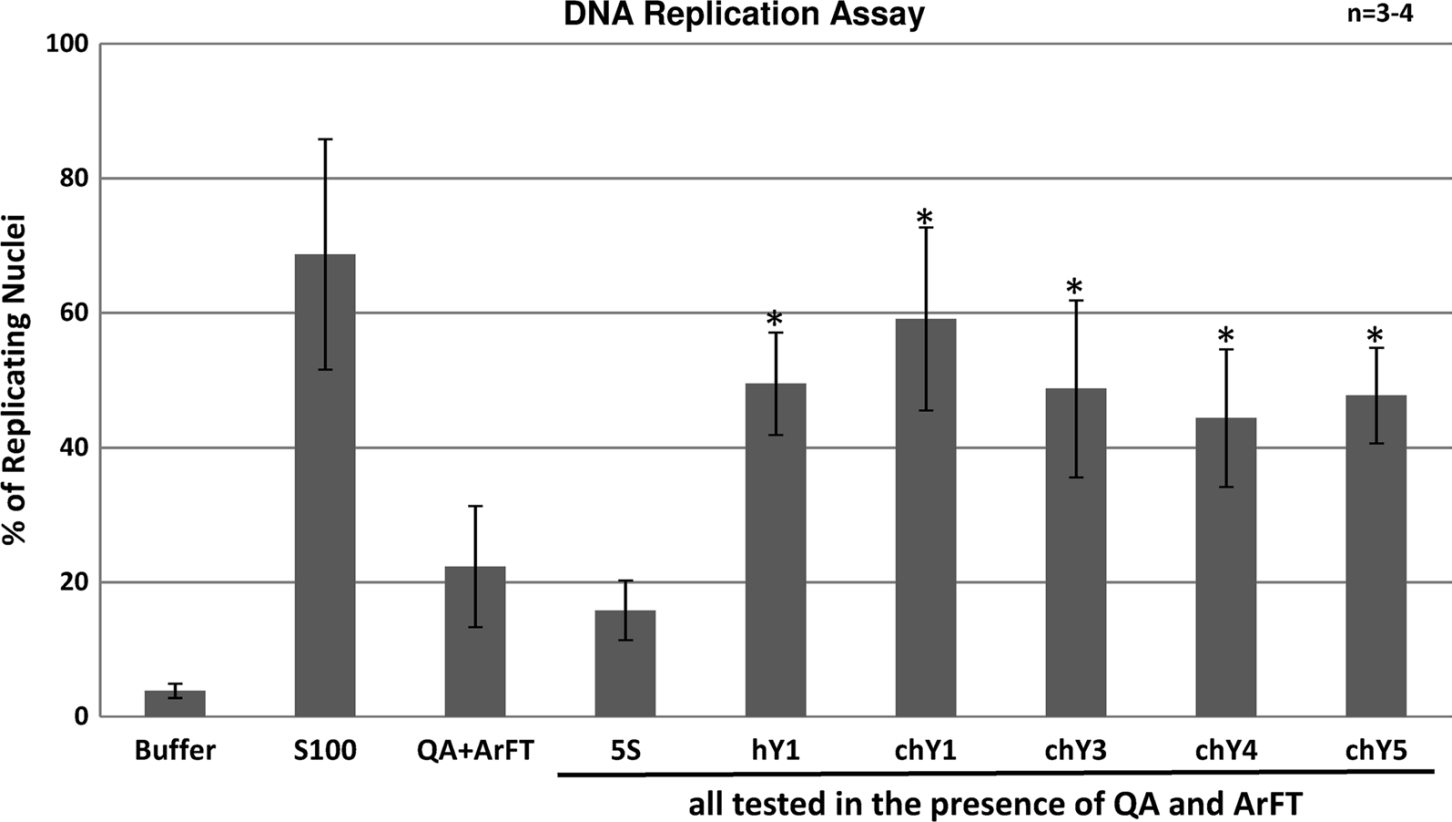
Chinese hamster Y RNAs can substitute for hY RNAs for the reconstitution of chromosomal DNA replication. Quantitative analysis of replicating G1-phase nuclei in vitro (see “Methods”). Human EJ30 nuclei were separately incubated with buffer, unfractionated cytosol of HeLa cells (S100), fractions QA and ArFT, and with fractions supplemented with 300 ng of the individual RNAs synthesized in vitro as indicated [8]. The human 5S ribosomal RNA was used as negative control. Mean values and standard deviations of the proportions of replicating nuclei from 3 to 4 independent experiments (n) are shown. *P < 0.05 (student’s *t* test) when compared to negative control reaction containing 5S rRNA

Next we analyzed if chY RNAs could functionally substitute for the hY RNAs in a human cell-free DNA replication system. In this system, a cytosolic extract from human proliferating cells initiates and supports bidirectional semiconservative DNA replication in more than 60 % of template nuclei, which are prepared from late G1 phase human cells [8, 30–34]. This extract contains endogenous hY RNAs and all essential soluble DNA replication proteins. In the absence of the cytosolic extract, DNA replication is observed only in approximately 5 % of the nuclei (Fig. 2). The endogenous hY RNAs can be depleted from the cytosolic extract by biochemical fractionation, yielding two protein fractions containing all essential initiation proteins (termed QA and ArFT). Incubation of template nuclei with these two fractions alone resulted in DNA replication in 20 % of the nuclei (Fig. 2), likely due to small amounts of residual Y RNAs remaining from the fractionation of the cytosolic extract [8]. As shown before [8], addition of purified exogenous hY1 RNA increased the proportion of replicating nuclei to about 40 %, whereas addition of human ribosomal 5S rRNA as a negative control did not increase the proportion of replicating nuclei in this system (Fig. 2). Next, we synthesized all four chY RNAs in vitro and tested whether they could substitute for hY1 RNA in this assay. Indeed, each of the four chY RNAs significantly increased the proportion of nuclei replicating their chromosomal DNA over the negative control, 5S rRNA (t test, P < 0.05), and to the same extent as hY1 RNA (Fig. 2) Therefore, chY RNAs can substitute for hY RNAs to initiate and support DNA replication in vitro.

It is known that vertebrate Y RNAs can functionally substitute for human Y RNAs in chromosomal DNA replication in a cell-free system [9], and that human Y RNAs can replace mouse Y RNAs in a mouse cell-free DNA replication initiation system [4]. Our analysis reported here therefore consolidates the functional conservation of vertebrate Y RNAs and extends it to the Chinese hamster.

Although the expression analysis reveals the absence of detectable chY4 and chY5 RNAs in GMA32 Chinese hamster cells (Table 1), both RNAs were able to initiate DNA replication in vitro (Fig. 2). This is probably because these silent chY RNAs still feature functional motifs of Y RNAs (Fig. 1c, d, [9]). In a systematic mutagenesis screen, the upper stem of Y RNAs was shown to be essential and sufficient for chromosomal DNA replication in vitro and in vivo, showing that this domain is a key determinant for Y RNA function [9]. For the nucleotide sequences and predicted secondary structures of chY RNAs, the upper stem of chY4 RNA shows the greatest divergence from the consensus. It maintains the base-paired nucleotide GUG-CAC consensus motif essential for DNA replication, but flanking sequences are no longer predicted to form a base-paired double-stranded RNA helix (Fig. 1). To see if this domain may still assume an overall stable helix-like structure, which might be important to its observed DNA replication function, we conducted molecular dynamics simulations under physiological conditions. We sought to evaluate the stability of chY4 RNA compared to hY4 RNA, focusing on the upper stem GUG-CAC base pairs.

### Molecular modeling and dynamics of Y4 RNAs

**Fig. 3:**
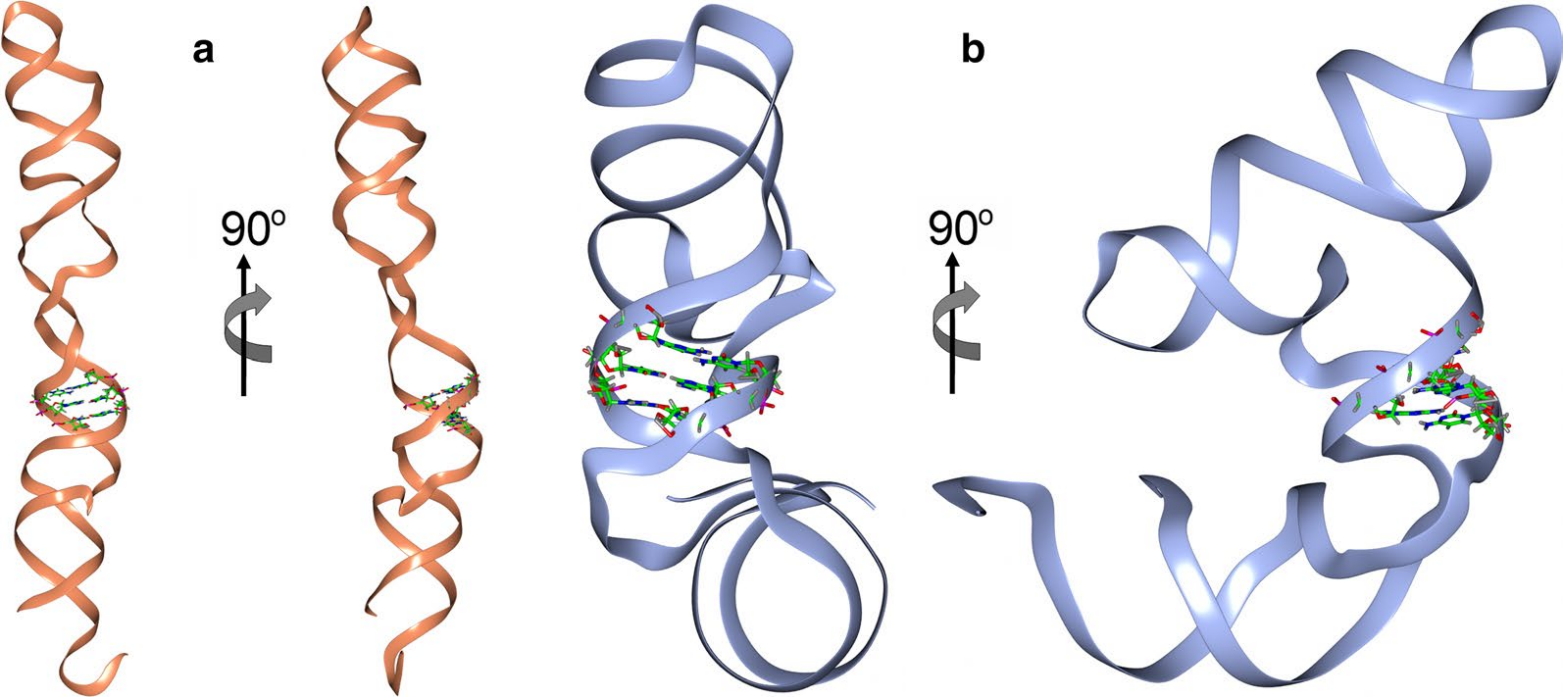
Ribbon model of Y4 RNAs. The illustration highlights the location of the functional motif GUG-CAC in the predicted 3D ribbon model structures of (**a**) hY4 RNA and (**b**) chY4 RNA

We generated predicted three-dimensional structures from the primary nucleotide sequences of human and Chinese hamster Y RNAs and generated pdb files for visualization (Additional file 4: Files S1), using the RNA Composer server [35]. In the three-dimensional ribbon band representations of the two Y4 RNAs, the conserved and functionally essential GUG-CAC trinucleotide motifs are located in an exposed and extended region of the molecules, which could facilitate an interaction with a cellular target (Fig. 3).

**Fig. 4:**
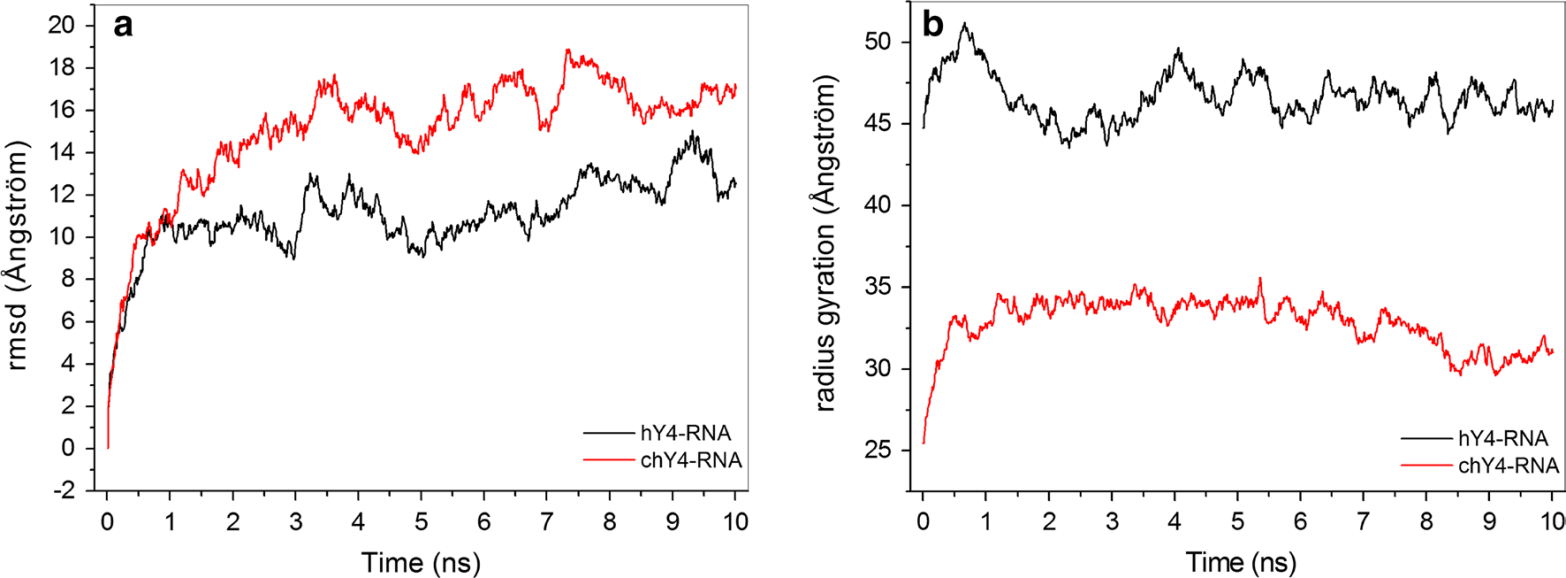
Simulation behavior of the Y4 RNAs. The trajectory of the molecular dynamics was analyzed from C1′ of Y4 RNAs in terms of root mean square deviation (**a**) and radius of gyration (**b**). The simulation was carried out with periodic boundary conditions at 300 K temperature, 1 atm pressure, pH 7.0, NaCl 0.1 M

These 3D structures of Y4 RNAs were then used for simulations of equilibration molecular dynamics, and the analysis of their behavior was performed from the trajectory file. Figure 4a shows the root mean square deviation (RMSD) calculated from the C1′ carbon of each nucleotide. These results provide evidence that both hY4 and chY4 RNAs reached equilibrium already after 3 ns of simulation time. The molecular dynamics simulations are usually performed for a longer period of time [36], however, and Fig. 4a shows that 10 ns was a sufficient time to have an overall picture of the system in balance. Figure 4b shows the radius of molecular gyration of the two Y4 RNAs during the simulation. The chY4 RNA oscillates around 32.8Å ± 1.54 while the hY4 RNA oscillates around 46.6 Å ± 1.39. This result indicates that the segments of both RNA models representing the C1′ carbon of each nucleotide keep their original fold design over simulation time, indicative of stable structures for both hY4 and chY4 RNAs.

**Fig. 5:**
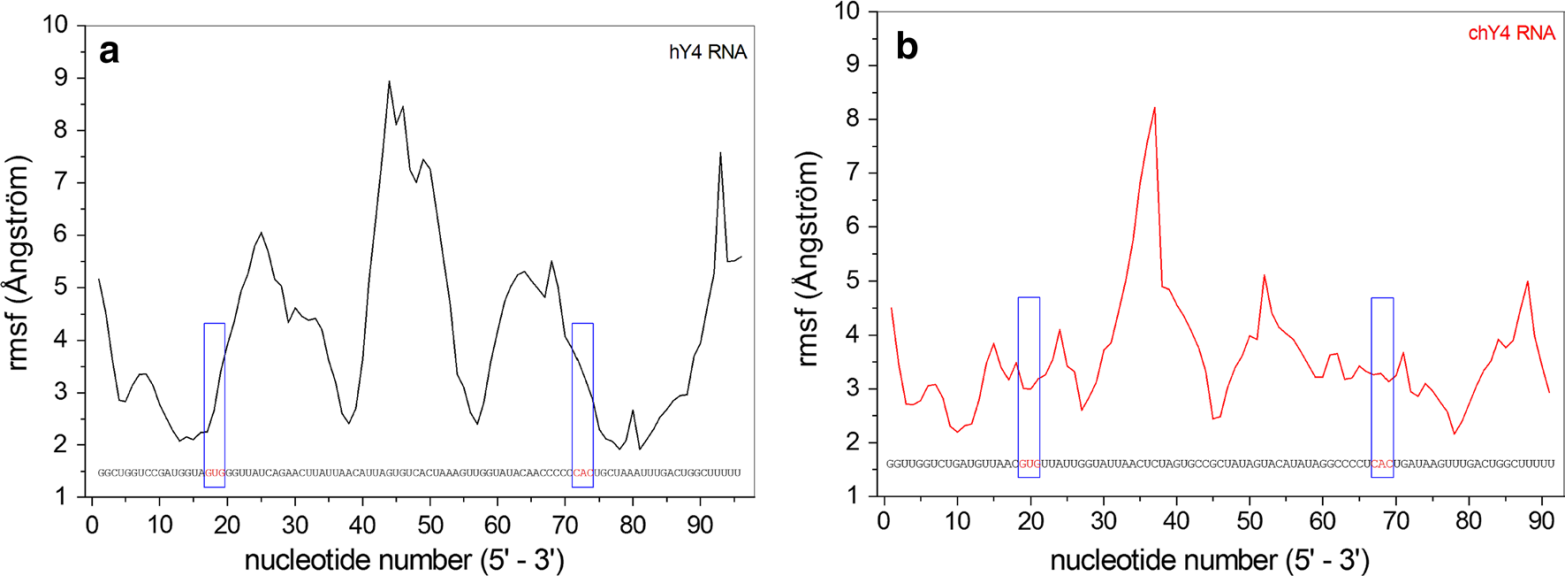
Fluctuation of C1′ atoms of each nucleotide from Y4 RNA segments. **a** hY4 RHA and **b** chY4 RNA. The blue squares highlight the root mean square fluctuation of the functional motif GUG-CAC showing that these triplets fluctuate much less than other regions of the molecule

**Fig. 6:**
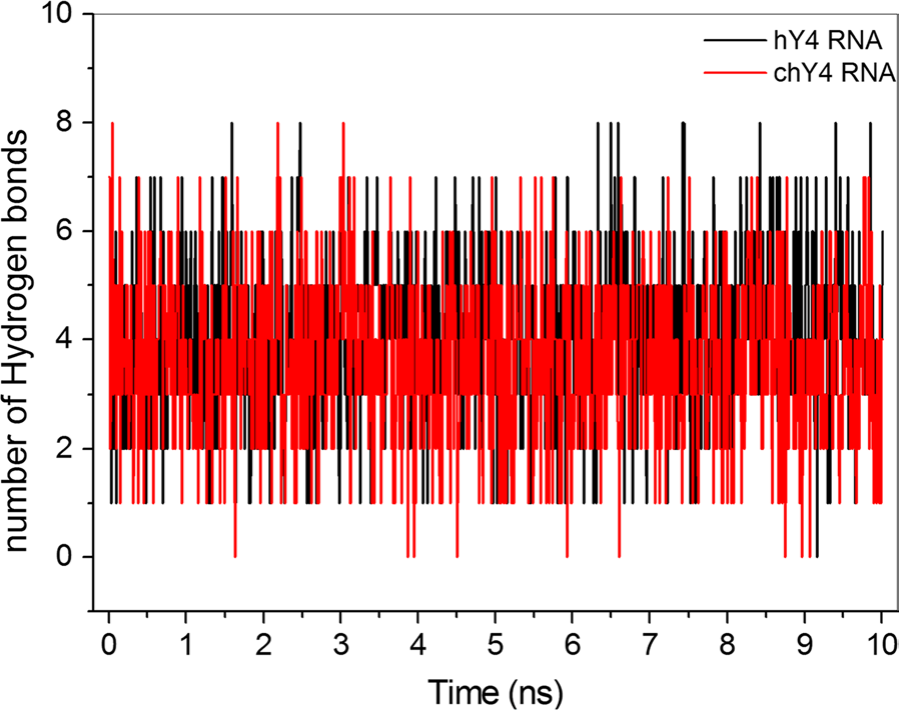
Hydrogen bonds between the functional motif GUG-CAC of Y4 RNAs. The number of bonds remained constant throughout the simulation, which means that these homologous regions are equally stable in both structures

The root mean square fluctuation (RMSF) for each nucleotide of hY4 and chY4 RNA is shown in Fig. 5. According to these simulations, the region of the functional GUG-CAC motif in the hY4 RNA showed an increasing RMSD value reaching 4.0 Å towards the open central loop domain (Fig. 5a). The homologous region in chY4 RNA, despite being located in a highly flexible region between two loops, presents a constant low RMSF value of approximately 3.5 Å (Fig. 5b), suggesting that this region appears to be a little more stable in chY4 than in hY4 RNA. Most of this stability is due to the Watson–Crick base pairing of both motifs, which tends to be more stable than the non-canonical G-U base pairing [37]. In addition, we observed a constant number of hydrogen bonds formed between the base pairs of GUG-CAC over the simulation time for both chY4 and hY4 RNAs (Fig. 6).

We conclude that the predicted structural stability of this functional double-stranded region in chY4 RNA could be relevant for the interaction of Y RNAs with their cellular targets [38–40] and would allow this RNA to perform its function in DNA replication like other vertebrate Y RNAs. We have recently investigated the solution structure of the corresponding domain in hY1 RNA and obtained biophysical evidence for a dynamic structure of the conserved GUG-CAC motif, which is embedded in a stable A-form helix representing the entire upper stem domain [15]. Future experiments will be required to characterize the functional interaction of this conserved domain with relevant binding proteins or chromatin structures in the vicinity of DNA replication origins.

## Conclusions

In this study, we have identified that the Chinese hamster genome contains four individual genes for chY RNAs (*chY1*, *chY3*, *chY4* and *chY5* RNA). Although these genes are homologous to the four human *hY* RNA genes, only the two *chY1* and *chY3* RNA genes, which have the highest level of homology to the corresponding human genes, are expressed. The predicted structures of these Chinese hamster RNA molecules show a high level of conservation with the hY RNAs, except for chY4, but the molecular dynamics simulations suggest that the three-dimensional structures of both chY4 and hY4 RNAs should be stable under physiological conditions. Functional testing of the chY RNAs established that they can substitute for hY RNAs to initiate the DNA replication in vitro. In conclusion, the results of this work establish that the structure and function of vertebrate Y RNAs extends to the Chinese hamster, which now allows future investigations of chY RNA-dependent regulation of DNA replication in the interesting case of the replication origins of the AMPD2 amplicon.

## Methods

### Homology search and structure modeling

All human Y RNAs were independently used for the BLASTn searches in the Chinese hamster genome. When the homology search provided us with any sequence of Y RNAs with relatively high homology, we searched for conserved structural features to be able to determine whether this represented a true gene and not a pseudogene. These required structural characteristics include the presence of an RNA polymerase III terminator (i.e., a stretch of Ts) and at least a TATA box as a promoter element located at the appropriate distance (−32 to −25 relative to the start of transcription) [27, 29]. Predicted secondary structures for all RNAs were calculated from the full-length nucleotide sequence using the Mfold v3.2 RNA folding algorithm under default conditions [41]. The downloaded Vienna files were used to draw 2D model structures using VARNA applet [42]. The 3D structures were generated as pdb files using the RNA Composer server (Additional file 4: Files S1) [35].

### Molecular dynamics simulations

For molecular dynamics simulation of Y4 RNAs, the three-dimensional models were virtually immersed in a periodic box containing SPC water and 100 mM NaCl with dimensions at least 15 Å away from the outermost surface of the molecule. Initially, all systems were minimized by the steepest descent method implemented in the program Gromacs-4.5.5 [43]. The final minimized structures were used as an input parameter for the equilibration molecular dynamics using the AMBER99SB force field [44], one of the most well-established simulation codes for nucleic acids [45]. Simulations were carried out during 10 ns, temperature 300 K and 1 atm of pressure. All other parameters were adjusted for default conditions of the Gromacs-4.5.5 program. All analyses were performed on the ensemble of system configurations extracted at 2 ps time intervals from the simulations.

### Cell culture

The GMA32 cell line, which was generously provided by Dr. Michelle Debatisse (Institute Curie, Paris, France), is a deoxycytidine kinase (dCK) deficient derivative of the CCL39 line of Chinese hamster lung fibroblasts. Tissue culture was performed as previously described [16]. No animals have been used in this work.

### Molecular cloning and sequencing

To perform the molecular cloning of chY RNAs, the genomic DNA from GMA32 cell line has been extracted using the AxyPrep™ Multisource Genomic DNA (Axygen^®^) kit. Full-length DNA sequences encoding for chY RNAs were generated by PCR amplification using genomic DNA as a template. All forward primer sequences contained a 5′ SP6 promoter site as previously described (Additional file 5: Table S1) [8]. The PCR products for all four chY RNAs were cloned into the TOPO^®^ TA Cloning^®^ kit (Invitrogen). The transformation was performed in competent DH5α bacteria through heat shock [46]. The plasmid purification from selected clones was performed using the CTAB method [47]. The selected clones were amplified by PCR using the M13 primer pair. The sequencing has been carried out using the DYEnamic ET Terminator (Amersham Biosciences) kit in Molecular Dynamics MegaBACE 1000 DNA Analysis System [48].

### Expression and purification of recombinant Y RNAs

Recombinant Y RNAs were synthesized by in vitro transcription using SP6 RNA polymerase as previously described [8, 9]. RNAs were purified by anion exchange chromatography on a MonoQ column (Amersham Biosciences) as previously described [49]. The size and purity of all in vitro-synthesized RNA were confirmed using 8 M urea denaturing polyacylamide gel electrophoresis and staining with SYBR Gold (Invitrogen) as described [8].

### DNA replication in vitro

Cell culture, cell synchronization, preparation of template nuclei, extract fractionation, DNA replication in vitro, and analysis of DNA replication reactions were performed as previously described [8, 9, 30–32]. In this study, nuclei were prepared from human EJ30 bladder carcinoma cells and cell extracts were prepared from HeLa cells.

### Quantitative real-time PCR

For analysis of chY RNAs expression in the GMA32 cell line, cDNA was synthesized from total RNA using random primers. The cDNA mixture was used as a template for the quantitative real-time PCR reaction (qPCR), which was performed in the iCycler iQ™ device, using the SYBR green supermix labeling kit (Bio-Rad) over 40 cycles and a hybridization temperature of 55 °C, as previously described [8]. For RNA-specific cDNA amplification, the primer sequences are provided in Additional file 6: Table S2.

## Availability of supporting data

The data sets supporting the results of this article are included within the article and its additional files

## Additional files

10.1186/s12867-015-0053-5 Sequence alignment between human and Chinese hamster Y RNAs. The divergent nucleotides are indicated in yellow.
10.1186/s12867-015-0053-5 ClustalW multiple sequence alignment of promoter regions from *Y5* RNA gene of *Homo sapiens* (*hY5*), *Cricetulus griseus* (*chY5*), and *Mus musculus* (*mY5*). The type 3 promoter of RNA polymerase III comprises a distal sequence element (DSE, −215 to −240, in light grey) that enhances transcription and a core promoter composed of a proximal sequence element (PSE, −65 to −48, in green), and a TATA box (from −32 to −25, relative to the start site of transcription, in light blue). The Y5 RNA sequences are shown in yellow.
10.1186/s12867-015-0053-5 Phylogenetic tree showing the evolution of Y RNAs from *Homo sapiens* (*hY1* [NR_004391.1], *hY3* [NR_004392.1], *hY4* [NR_004393.1] and *hY5* [NR_001571.2]), *Mus musculus* (*mY1* [NR_004419.1], *mY3* [NR_024202.2] and *mY5* [putative sequence not published]), and *Cricetulus griseus* (*chY1* [JX559781.1], *chY3* [JX976178.1], *chY4* [JX976179.1], and *chY5.*[JX976180.1]).
10.1186/s12867-015-0053-5 Y RNAs files in the.pdb format.
10.1186/s12867-015-0053-5 Primers designed for chY RNAs cloning. The SP6 promoter sequence is shown in green.
10.1186/s12867-015-0053-5 Primers designed for chY RNAs expression analysis.

## Abbreviations

hY RNAs: human Y RNAs
chY RNAs: Chinese hamster Y RNAs
mY RNAs: mouse Y RNAs
Ro RNPs: Ro ribonucleoprotein particles
QA: Q sepharose fraction A
ArFT: arginine sepharose flow through
RPA: replication protein A
PCNA: proliferating cell nuclear antigen
RMSD: root mean square deviation
RMSF: root mean square fluctuation
CTAB: cetyltrimethylammonium bromide
