# Supplementary figures and images for: Structural and functional analysis of four non-coding Y RNAs from Chinese hamster cells: identification, molecular dynamics simulations and DNA replication initiation assays
Source: BMC Mol Biol. 2016 Jan 5;17:1. doi: 10.1186/s12867-015-0053-5 (PMC4702372; doi:10.1186/s12867-015-0053-5)

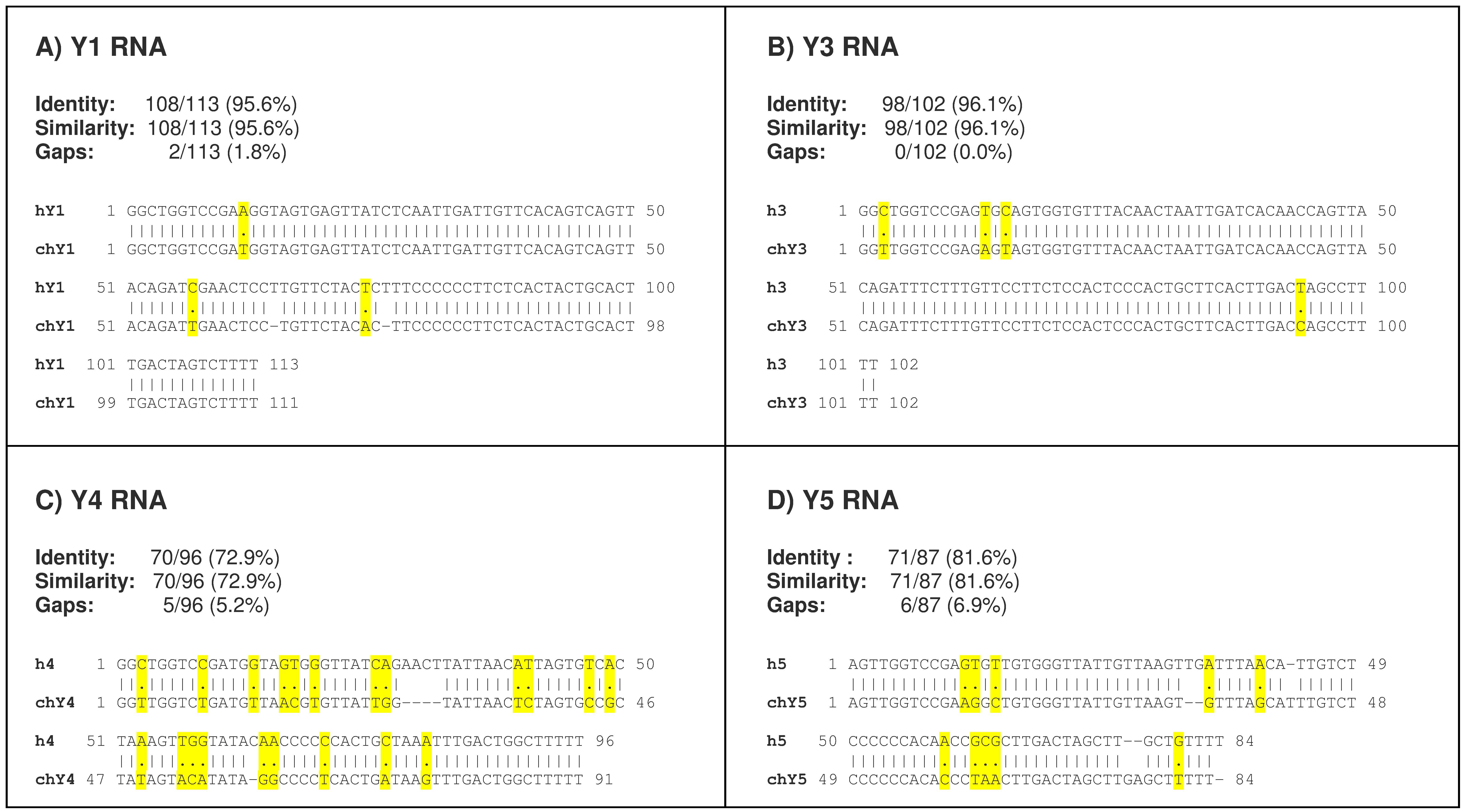

Supplement: Supplementary file 1 — 10.1186/s12867-015-0053-5 Sequence alignment between human and Chinese hamster Y RNAs. The divergent nucleotides are indicated in yellow. [file 12867_2015_53_MOESM1_ESM.tif]

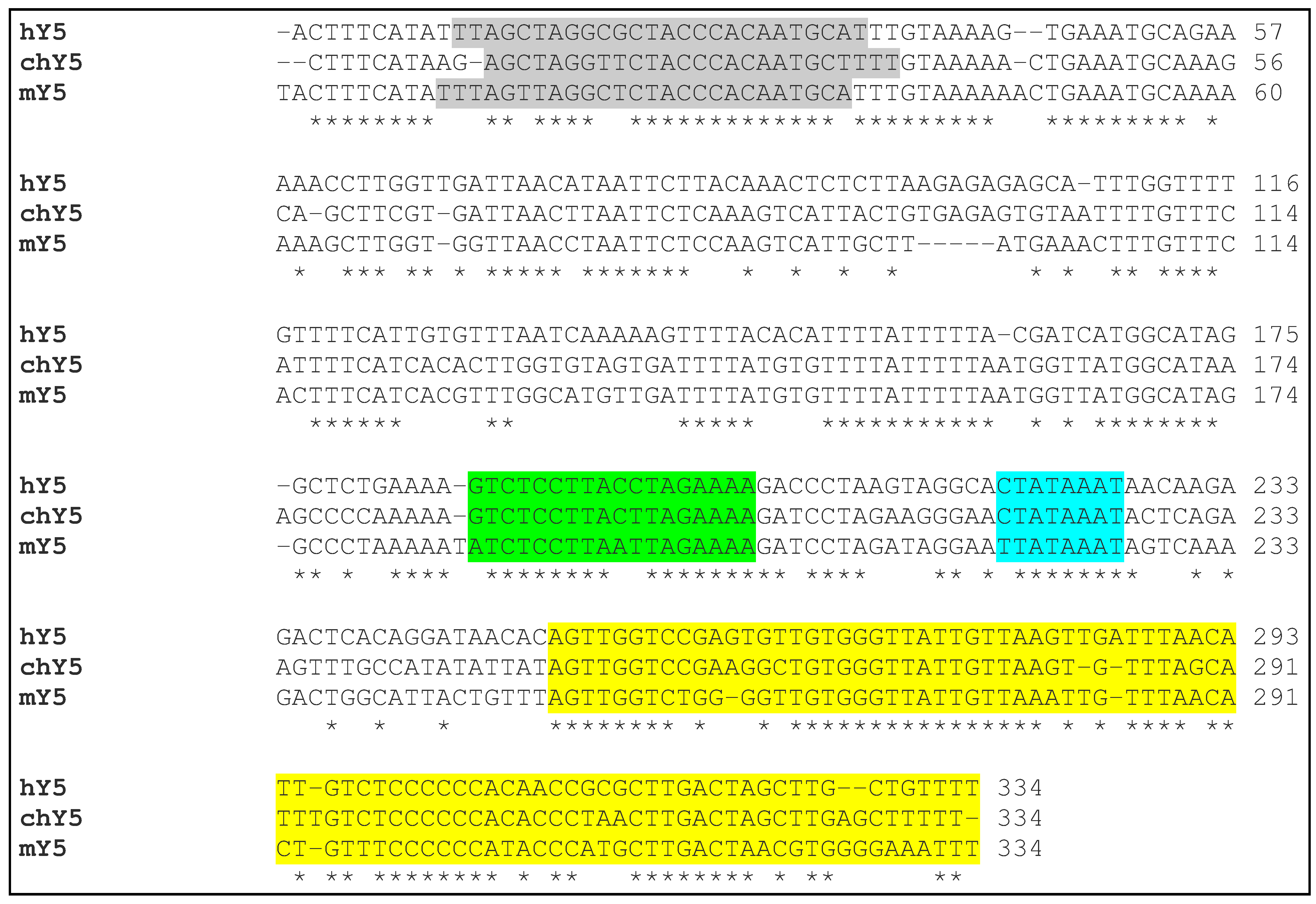

Supplement: Supplementary file 2 — 10.1186/s12867-015-0053-5 ClustalW multiple sequence alignment of promoter regions from Y5 RNA gene of Homo sapiens (hY5), Cricetulus griseus (chY5), and Mus musculus (mY5). The type 3 promoter of RNA polymerase III comprises a distal sequence element (DSE, −215 to −240, in light grey) that enhances transcription and a core promoter composed of a proximal sequence element (PSE, −65 to −48, in green), and a TATA box (from −32 to −25, relative to the start site of transcription, in light blue). The Y5 RNA sequences are shown in yellow. [file 12867_2015_53_MOESM2_ESM.tif]

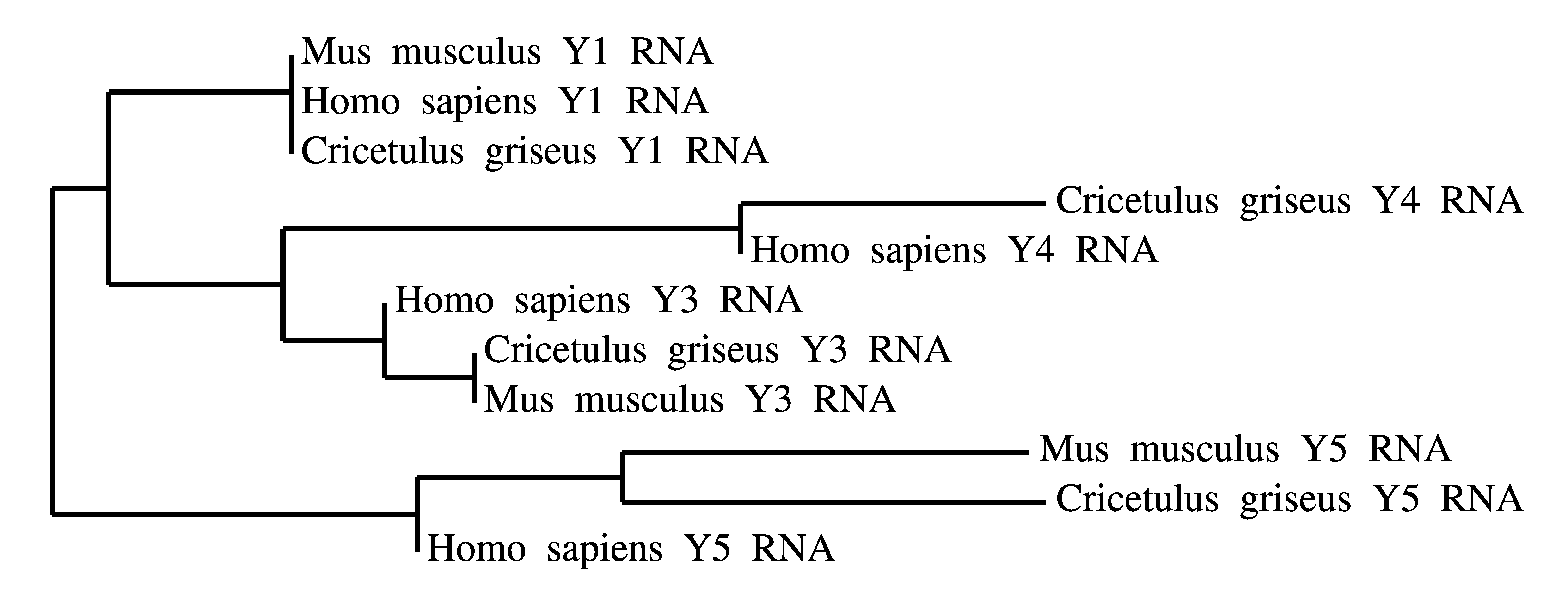

Supplement: Supplementary file 3 — 10.1186/s12867-015-0053-5 Phylogenetic tree showing the evolution of Y RNAs from Homo sapiens (hY1 [NR_004391.1], hY3 [NR_004392.1], hY4 [NR_004393.1] and hY5 [NR_001571.2]), Mus musculus (mY1 [NR_004419.1], mY3 [NR_024202.2] and mY5 [putative sequence not published]), and Cricetulus griseus (chY1 [JX559781.1], chY3 [JX976178.1], chY4 [JX976179.1], and chY5.[JX976180.1]). [file 12867_2015_53_MOESM3_ESM.tif]
