# Supplementary material for: Structural and functional analysis of four non-coding Y RNAs from Chinese hamster cells: identification, molecular dynamics simulations and DNA replication initiation assays
Source: BMC Mol Biol. 2016 Jan 5;17:1. doi: 10.1186/s12867-015-0053-5 (PMC4702372; doi:10.1186/s12867-015-0053-5)
Supplement: Supplementary file 5 — 10.1186/s12867-015-0053-5 Primers designed for chY RNAs cloning. The SP6 promoter sequence is shown in green. [file 12867_2015_53_MOESM5_ESM.docx]

| **chY RNA** | **Primer Sequences** |
| --- | --- |
| **chY1** | (F) 5'-ATTTAGGTGACACTATAGAAGGCTGGTCCGATGGTAGTGA |
|  | (R) 5'-AAAAGACTAGTCAAGTGCAG |
| **chY3** | (F) 5'-ATTTAGGTGACACTATAGAAGGTTGGTCCGAGAGTAGTGGTGTTTACAAC |
|  | (R) 5'-AAAAGGCTGGTCAAGTGAAGCAGTGGGAGTGGAGAAGGAACAAAG |
| **chY4** | (F) 5'-ATTTAGGTGACACTATAGAAGGTTGGTCTGATGTTAACGTGTTATTGGTA |
|  | (R) 5'-AAAAAGCCAGTCAAACTTATCAGTGAGGGGCCTATATGTACTATAGCGG |
| **chY5** | (F) 5'- ATTTAGGTGACACTATAGAAAGTTGGTCCGAAGGCTGTGGGTTATTGTTA |
|  | (R) 5'-AAAAAGCTCAAGCTAGTCAAGTTAGGGTGTGGGGGGAGACAAATGC |

**Additional file Table S1:** Primers designed for chY RNAs cloning. In green is showed the SP6 promoter sequence.
