# Supplementary material for: Structural and functional analysis of four non-coding Y RNAs from Chinese hamster cells: identification, molecular dynamics simulations and DNA replication initiation assays
Source: BMC Mol Biol. 2016 Jan 5;17:1. doi: 10.1186/s12867-015-0053-5 (PMC4702372; doi:10.1186/s12867-015-0053-5)
Supplement: Supplementary file 6 — 10.1186/s12867-015-0053-5 Primers designed for chY RNAs expression analysis. [file 12867_2015_53_MOESM6_ESM.docx]

| **chY RNA** | **Primer Sequences** |
| --- | --- |
| **chY1** | (F) 5'-GGCTGGTCCGATGGTAGTGA |
|  | (R) 5'-GCAGTAGTGAGAAGGGGGGA |
| **chY3** | (F) 5'-GGTTGGTCCGAGAGTAGTGG |
|  | (R) 5'-GAAGCAGTGGGAGTGGAGAA |
| **chY4** | (F) 5'-GGTTGGTCTGATGTTAACGTG |
|  | (R) 5'-TTATCAGTGAGGGGCCTATA |
| **chY5** | (F) 5'- AGTTGGTCCGAAGGCTGTGG |
|  | (R) 5'-AAGCTCAAGCTAGTCAAGTT |

**Additional file Table S2:** Primers designed for chY RNAs expression analysis.
